# Use of the FebriDx^®^ host-response point-of-care test may reduce antibiotic use for respiratory tract infections in primary care: a mixed-methods feasibility study

**DOI:** 10.1093/jac/dkae127

**Published:** 2024-05-06

**Authors:** Christopher R Wilcox, Nour Odeh, Tristan W Clark, Ingrid Muller, Taeko Becque, Alexander Todd, Nazrul Islam, Paul Little, Firoza Davies, John McGavin, Nick Francis

**Affiliations:** Primary Care Research Centre, Aldermoor Health Centre, Aldermoor Close, University of Southampton, Southampton SO16 5ST, UK; Primary Care Research Centre, Aldermoor Health Centre, Aldermoor Close, University of Southampton, Southampton SO16 5ST, UK; School of Clinical and Experimental Sciences, University of Southampton, Southampton General Hospital, Tremona Road, Southampton SO16 6YD, UK; NIHR Southampton Biomedical Research Centre, Southampton General Hospital, Tremona Road, Southampton SO16 6YD, UK; Primary Care Research Centre, Aldermoor Health Centre, Aldermoor Close, University of Southampton, Southampton SO16 5ST, UK; Primary Care Research Centre, Aldermoor Health Centre, Aldermoor Close, University of Southampton, Southampton SO16 5ST, UK; Lilliput Surgery, Shore Medical Group, Elms Avenue, Poole BH14 8EE, UK; Primary Care Research Centre, Aldermoor Health Centre, Aldermoor Close, University of Southampton, Southampton SO16 5ST, UK; Primary Care Research Centre, Aldermoor Health Centre, Aldermoor Close, University of Southampton, Southampton SO16 5ST, UK; Patient and Public Involvement Representative, Primary Care Research Centre, Aldermoor Health Centre, Aldermoor Close, University of Southampton, Southampton SO16 5ST, UK; Patient and Public Involvement Representative, Primary Care Research Centre, Aldermoor Health Centre, Aldermoor Close, University of Southampton, Southampton SO16 5ST, UK; Primary Care Research Centre, Aldermoor Health Centre, Aldermoor Close, University of Southampton, Southampton SO16 5ST, UK; NIHR Southampton Biomedical Research Centre, Southampton General Hospital, Tremona Road, Southampton SO16 6YD, UK

## Abstract

**Introduction:**

FebriDx^®^ is a CE-marked, single-use point-of-care test with markers for bacterial [C-reactive protein (CRP)] and viral [myxovirus resistance protein A (MxA)] infection, using finger-prick blood samples. Results are available after 10–12 min. We explored the usability and potential impact of FebriDx^®^ in reducing antibiotic prescriptions for lower respiratory tract infection (LRTI) in primary care, and the feasibility of conducting a randomized controlled trial (RCT).

**Methods:**

Patients (aged ≥1 year) with LRTI deemed likely to receive antibiotic prescription were recruited at nine general practices and underwent FebriDx^®^ testing. Data collection included FebriDx^®^ results, antibiotic prescribing plan (before and after testing) and re-consultation rates. Staff completed System Usability Scale questionnaires.

**Results:**

From 31 January 2023 to 9 June 2023, 162 participants participated (median age 57 years), with a median symptom duration of 7 days (IQR 5–14). A valid FebriDx^®^ result was obtained in 97% (157/162). Of 155 patients with available results, 103 (66%) had no detectable CRP or MxA, 28 (18%) had CRP only, 5 (3%) had MxA only, and 19 (12%) had both CRP and MxA. The clinicians’ stated management plan was to prescribe antibiotics for 86% (134/155) before testing and 45% (69/155) after testing, meaning a 41% (95% CI: 31%, 51%) difference after testing, without evidence of increased re-consultation rates. Ease-of-use questionnaires showed ‘good’ user-friendliness.

**Conclusions:**

Use of FebriDx^®^ to guide antibiotic prescribing for LRTI in primary care was associated with a substantial reduction in prescribing intentions. These results support a fully powered RCT to confirm its impact and safety.

## Introduction

Clinically differentiating bacterial from viral lower respiratory tract infection (LRTI) is challenging, with LRTI having the highest inappropriate antibiotic prescribing rates of conditions seen in primary care.^1^ Inappropriate antibiotic use risks side effects and drives antimicrobial resistance.^2^ Rapid diagnostic testing [‘point-of-care testing’ (POCT)] has potential to reduce antibiotic use,^3–6^ but its adoption into UK primary care remains limited.^7,8^

FebriDx^®^ (Lumos Diagnostics, USA)^9^ is a single-use, hand-held, lateral flow POCT device designed to help distinguish bacterial from viral infections. It detects two host-response proteins, c-reactive protein (CRP) and myxovirus resistance protein A (MxA), in finger-prick blood, with results available after 10–12 min. CRP is an acute phase reactant that generally increases to higher levels with bacterial compared to viral infection, and MxA is a derivative of interferon α/β associated with viral infection.^10,11^

As a dual-marker test, FebriDx^®^ may be more clinically useful than POCT devices detecting a single biomarker (typically CRP alone) or a specific pathogen (such as SARS-CoV-2). Furthermore, FebriDx^®^ does not require a separate desktop analyser, which may improve ease-of-use and reduces up-front costs and maintenance requirements.^3,5,6,12^ Studies in secondary care demonstrated good diagnostic accuracy compared to PCR.^13–19^ A recent study in the USA showed an agreement of 91.7% for bacterial detection (sensitivity 80%, specificity 93%) and 84% for viral detection (sensitivity 87%, specificity 83%).^19^ Several studies have investigated FebriDx^®^ as an emergency department triage tool (particularly for COVID-19), but there is limited data antibiotic prescribing or usability measures.^13,15,16,18–20^ Only one single-site retrospective study involving 21 patients has studied the impact of FebriDx^®^ in primary care.12

Further studies are needed in UK primary care to establish the impact on antibiotic use, in addition to usability, acceptability, safety, and cost-effectiveness. With a view to carrying out a future randomized controlled trial (RCT), the aims of this mixed-methods feasibility study were to explore:

- The usability and potential impact of FebriDx^®^ in reducing antibiotic use for LRTI in primary care.
- The feasibility of conducting a future RCT assessing the use of FebriDx^®^ in primary care.

## Methods

### Study design and setting

This was a prospective, mixed-methods, multi-centre, non-randomized, feasibility study, with an additional qualitative interview study (reported separately), coordinated by the University of Southampton Primary Care Research Centre. Data collection took place at nine general practice (GP) sites across South England. The study was pre-registered on clinicaltrials.gov (NCT05534555) and the protocol has been published.^21^ We followed the CONSORT guidelines for pilot and feasibility trials.^22^

### Patient and GP surgery recruitment

All research-active GP surgeries in South England under the Wessex NIHR Clinical Research Network (CRN) were invited and eligible to take part. Participating practices received financial compensation in line with National Institute for Health and Care Research guidance. Prescribing clinicians assessed patient eligibility, however, any appropriately trained healthcare professional could take informed consent and perform FebriDx^®^ testing. Training was provided by the study team, and staff were observed performing practice tests to ensure competence before proceeding with the study.

### Eligibility criteria

Patients (aged  ≥1 year) presenting to their GP surgery remotely or in-person with symptoms suggestive of a LRTI were eligible following clinical assessment if a prescribing clinician deemed that they would be likely to prescribe antibiotics in the absence of further diagnostic testing. We defined suspected LRTI as a cough, lasting <21 days, judged to be infective in origin, with other symptoms or signs localizing to the lower respiratory tract (shortness of breath, sputum, chest pain).^23^ Antibiotic prescriptions could be immediate or delayed (advised to wait for a specified period before taking them, and only if necessary). Patients were ineligible if they had taken antibiotics in the last 30 days or were unwilling/unable to provide informed consent.

### Intervention

**Figure 1.**
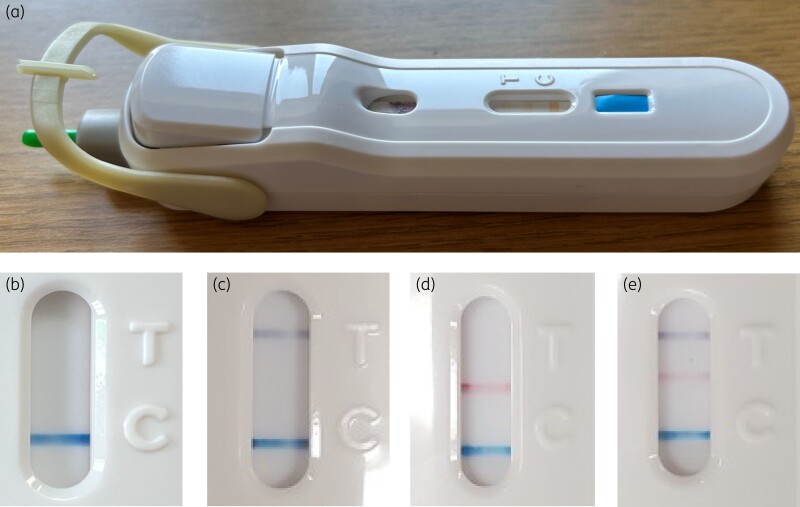
The FebriDx^®^ device and its possible results. (a) The FebriDx^®^ device. (b) Negative result with control line. (c) CRP-only positive. (d) MxA-only positive. (e) CRP and MxA positive. Figures 1b-e supplied by Lumos Diagnostics, USA. This figure appears in colour in the online version of *JAC* and in black and white in the print version of *JAC*.

FebriDx^®^ (Lumos Diagnostics, USA)^9^ is a CE-marked, FDA-approved, single-use POCT device with a turnaround time of 10–12 min (Figure 1). Capillary blood obtained by finger-prick (5 μL) of is drawn into a sample tube, transferred to a lateral flow strip, and test reagents released with a button. Results are generated in the form of three lines: a grey line indicating elevated CRP (lower limit of detection = 20 mg/L), a red line indicating elevated MxA (lower limit of detection = 40 ng/ml), and a blue control line indicating a valid test. An elevated MxA, with or without elevated CRP, is suggestive of viral infection. The presence of elevated CRP alone is suggestive of a bacterial infection. Presence of a control line only indicates a negative test result for both markers.

Verbal and written guidance was provided during study training. Practices were given flexibility over how to integrate the FebriDx^®^ into their clinics. In the case of a failed test, participants were offered repeat testing. Once results were available, these were interpreted by the recruiting clinician and communicated to the patient before proceeding with any clinical management deemed appropriate. Clinicians were advised to provide clear safety-netting advice regarding the need to seek medical attention in the event of persistent or worsening symptoms.

Optional nasopharyngeal swabbing was introduced part-way through the study. Those who consented to this aspect were asked to provide a swab (taken by the staff or patient) that was then posted to Southampton General Hospital microbiology laboratory. Nasopharyngeal swabs were frozen on arrival, and later underwent viral analysis by multiplex PCR.

### Data collection

Data were collected by participating staff via an online case-report form completed in a sequential fashion before and after FebriDx^®^ testing. Initial pre-test data included baseline patient characteristics, clinical features of the presenting illness, clinicians’ perception of the likelihood of bacterial aetiology (graded 1–10 on a Likert scale, with 10 = ‘very likely’), and clinicians’ antibiotic prescription plan had no further testing been available (immediate, delayed, or no antibiotics).

Post-test data included FebriDx^®^ test result, the time of collection/result/when the patient was informed, clinicians’ post-test perception of the likelihood of bacterial aetiology on the same 10-point Likert scale, clinicians’ post-test antibiotic prescription plan, and clinicians’ post-test confidence in the need for antibiotics (graded 1–5 on a Likert scale, with 5 = ‘very confident that antibiotics ARE needed’). Follow-up data (after 28 days) included subsequent healthcare contacts, antibiotic prescriptions, and serious complications (including sepsis or death). Practice-level data collected included socioeconomic status (Index of Multiple Deprivation).

At the end of the study, practice staff were invited to complete an anonymous online ease-of-use questionnaire regarding the use FebriDx^®^. This contained the System Usability Scale (SUS), a well-established usability score that involved grading FebriDx^®^ on a 5-point Likert scale across 10 usability criteria^24^ (Figure S1, available as Supplementary data at *JAC* Online).

### Sample size

As a feasibility study, a formal sample size calculation was not required.^22^ With regards to antibiotic use, we calculated that 156 participants would allow us to describe feasibility or outcome rates of 50% to within a 95% confidence interval of ±7.8%. Rates higher or lower than 50% would be described with a greater precision.

### Data analysis

Statistical analysis was performed using STATA v.18 (StataCorp, USA, 2023). As this was a feasibility study, descriptive statistics are reported. Comparison of FebriDx^®^ with viral PCR was used to assess diagnostic accuracy (sensitivity and specificity). The analysis was conducted by C.W. with oversight from N.F./T.B./N.I.

## Results

**Figure 2.**
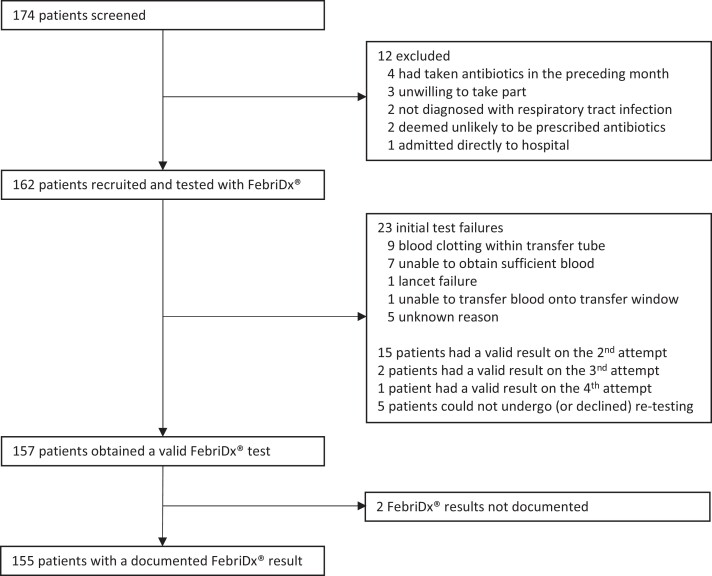
Flow diagram for the study.

From 31 January to 9 June 2023, 174 patients were screened, and 162 participants (93%) were recruited. Flow of study participants is displayed in Figure 2. Nine GP surgeries recruited a median of seven patients (IQR 5–28.5) (Table S1). Baseline demographics and clinical characteristics are displayed in Table 1. Median age was 57 years (IQR 40–69), 91% (147/162) were adults (median age 57 years, IQR 44–70) and 9% were children (median age 6 years, IQR 3–15). Sex was evenly balanced, but was not recorded in 28 (17%). Median symptom duration was 7 days (IQR 5–14).

**Table 1.** Baseline demographics and clinical characteristics of patients

	All patients, *n* = 162
Age, years (median, IQR)	57 (40–69)
0–5	7/162 (4%)
6–17	7/162 (4%)
8–64	93/162 (58%)
65–79	48/162 (30%)
80+	6/162 (4%)
Unknown	1/162 (1%)
Sex	
Male	67/162 (42%)
Female	68/162 (42%)
Unknown	27/162 (17%)
Ethnicity	
White British	149/162 (92%)
White Other	5/162 (3%)
Asian/Black/Mixed/Other	5/162 (3%)
Unknown	3/162 (2%)
Smoking status	
Current smoker	18/162 (11%)
Ex-smoker	59/162 (36%)
Never smoked	83/162 (51%)
Unknown	2/162 (1%)
Comorbidities	
Pregnancy	0/162 (0%)
Cardiovascular disease	32/162 (20%)
Respiratory disease	57/162 (35%)
Chronic kidney disease	4/162 (2%)
Diabetes mellitus	13/162 (8%)
Malignancy (active)	2/162 (1%)
Immunosuppression	3/162 (2%)
Hospital admission in previous 12 months	
None	140/162 (88%)
Unplanned for respiratory infection	5/162 (3%)
Unplanned for other reason	9/162 (6%)
Planned admission	5/162 (3%)
Vaccinations in previous 12 months	
Influenza	87/162 (54%)
SARS-CoV-2	95/162 (59%)
Symptoms at presentation	
Duration of symptoms, days (median, IQR)	7 (5–14)
Symptoms ≤7 days	87/162 (54%)
Symptoms >7 days	65/162 (40%)
Unknown	10/162 (6%)
Cough	
Mild	10/162 (6%)
Moderate	120/162 (74%)
Severe	30/162 (19%)
Unknown	2/162 (1%)
Productive cough	139/162 (86%)
Dyspnoea	
None	31/162 (19%)
Mild	63/162 (39%)
Moderate	58/162 (36%)
Severe	10/162 (6%)
Coryza	64/162 (40%)
Observations at presentation	
Temperature ≥38°C	7/107 (7%)
Hypoxia	7/126 (6%)
Tachycardia	17/124 (14%)
Tachypnoea	9/77 (12%)

### Test results and time to result

A valid test result was obtained in 97% (157/162) of participants: on the first attempt in 86% (139/162), on the second attempt in 10% (15/162), on the third or fourth attempt in 2% (3/162), and was abandoned in 3% (5/162). Reasons for initial test failure are displayed in Figure 2, and were most commonly due to difficulty obtaining sufficient blood from the fingerpick, or insufficient filling of the blood transfer tube. For two participants, the clinician documented that they obtained a valid test result, but did not document the result. Therefore, test results were available for 96% (155/162).

FebriDx^®^ results were available to interpret after a median of 10 min (IQR 10–11, *N* = 153), and patients were informed after a median of 2 minutes (IQR 0–5, *N* = 142), with a median total time of 13 min (IQR 10–15, *N = *142) from fingerpick to being informed. No CRP or MxA line (a negative result) occurred in 67% of cases (103/155), a CRP line only in 18% (28/155), a MxA line only in 3% (5/155), and both CRP and MxA lines in 12% (19/155). Negative results were more common (72% versus 64%) in those with symptoms for >7 days (Table 2).

**Table 2.** FebriDx^®^ results in all patients, and those with a symptom duration of ≤7 and >7 days

FebriDx^®^ result	All patients	Symptom duration ≤7 days	Symptom duration >7 days
Negative	103/155 (67%)	54/84 (64%)	44/61 (72%)
CRP only	28/155 (18%)	13/84 (16%)	11/61 (18%)
MxA only	5/155 (3%)	4/84 (5%)	1/61 (2%)
Both CRP and MxA	19/155 (12%)	13/84 (15%)	5/61 (8%)
TOTAL	155/155	84/145^a^	61/145^a^

^a^Ten of the 155 patients with a documented FebriDx^®^ result did not have data recorded on symptom duration.

### Pre- and post-test clinical impression and antibiotic management plan

Clinicians’ median grading of the likelihood of bacterial aetiology was 6/10 (IQR 4–7, *N* = 155) before testing and 3/10 after testing (IQR 1–6, *N = *154), with one patient having missing data.

Clinicians’ stated management plan was to prescribe immediate or delayed antibiotics for 86% (134/155) of participants before FebriDx^®^ testing and 45% (69/155) after testing, meaning there was a 41% (95% CI: 31%, 51%) difference before and after testing (Table 3). Following testing, 47% (73/155) had an antibiotic treatment plan that was likely to reduce antibiotic use (change from immediate antibiotics to none or delayed), 45% (70/155) had no change in their treatment plan, and 8% (12/155) had a change that would likely result in increased use (Table S2).

**Table 3.** Antibiotic prescription plan before and after FebriDx^®^ testing

		Post-test prescribing plan		
		Immediate antibiotics	Delayed antibiotics	No antibiotics
Pre-test prescribing plan	Immediate antibiotics (76/155, 49%)	41/76 (54%)	6/76 (8%)	29/76 (38%)
	Delayed antibiotics (58/155, 37%)	10/58 (17%)	10/58 (17%)	38/58 (65%)
	No antibiotics (21/155, 14%)	1/21 (5%)	1/21 (5%)	19/21 (90%)

**Figure 3.**
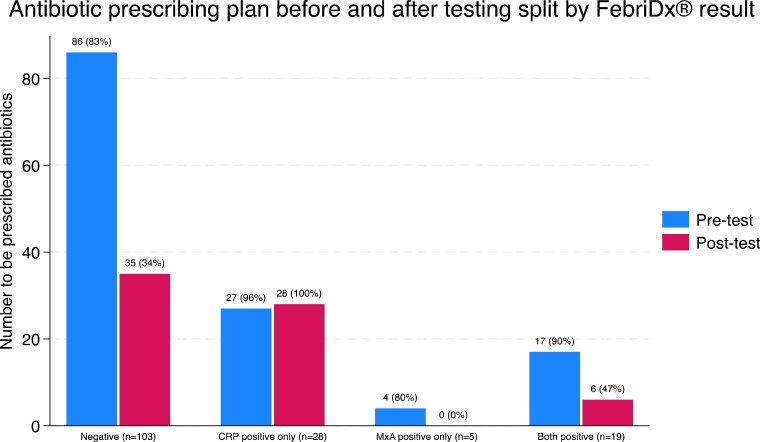
Antibiotic prescribing plan before and after testing split by FebriDx^®^ result. This figure appears in colour in the online version of *JAC* and in black and white in the print version of *JAC*.

Only those with a CRP-positive only result were more likely to receive antibiotics after testing, with all other results being associated with a reduction in antibiotic use (Figure 3). Clinicians indicated that they planned to prescribe antibiotics to 34% (35/103) of participants with a negative test result, 100% (28/28) with a CRP-only positive result, 0% (0/5) with a MxA-only result, and 32% (6/19) with a combined CRP/MxA positive result.

Clinicians reported increased confidence in their prescribing decisions in 82% (126/154) of cases (Table 4). Clinicians were more confident that antibiotics were not required in 51% (78/154), no difference in 18% (28/154), and more confident that antibiotics were required in 31% (48/154) (Figure S2).

**Table 4.** Clinician’s confidence in the need for antibiotics following FebriDx^®^ testing, split by FebriDx^®^ result

FebriDx^®^ result	More confident antibiotics NOT needed	No difference	More confident antibiotics needed
All patients (*N* = 154)^a^	78/154 (51%)	28/154 (18%)	48/154 (31%)
Negative (*N* = 102)	63/102 (62%)	24/102 (24%)	15/102 (15%)
CRP only (*N* = 28)	1/28 (4%)	2/28 (7%)	25/28 (89%)
MxA only (*N* = 5)	4/5 (80%)	0/5 (0%)	1/5 (20%)
Both CRP and MxA (*N = *19)	10/19 (53%)	2/19 (11%)	7/19 (37%)

^a^One of the 155 patients with a documented FebriDx^®^ result did not have data recorded on clinician confidence.

### Follow-up data on antibiotic use and re-attendance

Follow-up data were obtained via clinical notes review (after 28 days). The clinical records differed from the study case-report form on three occasions: one where a patient to be prescribed immediate antibiotics was admitted directly to hospital; and two where the initial management plan following a telephone review was changed after a planned face-to-face review with a GP later the same day (one prescribed immediate antibiotics rather than delayed antibiotics, and one prescribed no antibiotics rather than delayed antibiotics).

Twenty-three percent (35/155) sought additional medical attention for the same illness within 28 days of their initial consultation (Table S3). No serious adverse events were recorded. The highest re-consultation rate was seen among those prescribed immediate antibiotics (33%, 17/52). Furthermore, re-attendance rates were higher among patients for whom the clinician kept to their pre-test decision to prescribe immediate antibiotics (32%, 13/41), compared with patients for whom the clinician changed their decision from immediate antibiotics to delayed (0%, 0/6) or no antibiotics (28%, 8/29) following testing (Table S4). Antibiotics were prescribed after re-consultation in 15% (23/155) of cases, of whom 43% (10/23) had not been prescribed antibiotics initially, meaning the overall antibiotic prescription rate within 28 days was 51% (79/155).

### Viral PCR analysis and diagnostic accuracy

As a result of the late introduction of this voluntary aspect of the study we only obtained nasopharyngeal swab results for 18% (28/155) of participants and did not have sufficient data to report reliable test characteristics (Tables S5 and S6).

### Ease-of-use questionnaires

System usability score (SUS) questionnaires were returned from 89% (16/18) of GP practice staff who used FebriDx^®^ devices in the study, and at least one member from all sites. The mean SUS of 72.2 suggests a ‘good’ level of user-friendliness on a proposed adjective rating scale based on previous usability studies using the SUS.^24^

Further comments (Table S7) were provided by 38% (6/16) of respondents. Users were generally positive about the device, but acknowledged there was a ‘learning curve’ to its use. Additional specific points included practical difficulties with transferring blood from the collection tube onto the lateral flow test strip, difficulties interpreting results due to the faintness of result lines and the need for an even quicker turnaround time for it to be practical to integrate into a routine GP consultation.

## Discussion

This is the largest study evaluating the potential clinical impact of FebriDx^®^ in primary care, and demonstrates that FebriDx^®^ testing may reduce unnecessary antibiotic use in patients with LRTI. Following FebriDx^®^ testing, clinicians felt more confident in their antibiotic prescribing decisions more than 80% of the time, with more confidence that antibiotics were not required in over 50%. In keeping with this, their plan to prescribe antibiotics reduced from 86% of participants before testing to 45% following testing. Ease-of-use assessment demonstrated good user-friendliness, but identified some technical challenges and the need for operators to become skilled in using the device.

Strengths of the study were that it was a multi-centre prospective study that collected data on clinician intention, confidence and actual behaviour, as well as participant re-consultation, subsequent antibiotic use and outcomes. Exceeding our recruitment target meant an adequate sample size to address our feasibility outcomes. The main limitation is the lack of randomization, which limits our ability to conclude that the reduction in antibiotic use was caused by use of the FebriDx^®^ test, as clinicians may not in reality have acted according to their stated pre-test prescribing plan. Additionally, the feasibility nature of the study meant that we were limited to descriptive statistics. Nevertheless, the strength and consistency of signal seen is highly suggestive of an important effect. Other limitations include the relatively short run-in period, a low number of children and an uneven distribution of participants. These increase the potential for selection bias and reduce the generalisability of our results, however our qualitative interview sub-study (reported separately) does explore aspects of the usability/feasibility in more depth. The study had low ethnic minority representation and nearly all GP practices were in areas of high socioeconomic status (Index of Multiple Deprivation decile 9 or 10). The low number of viral swabs prevents us from providing data on diagnostic accuracy.

Several studies highlight the potential for POCT to reduce antibiotic use,^3–6^ however, most devices detect a single biomarker or pathogen, and actual uptake into UK primary care remains low.^7,8,25–27^ A recent meta-analysis of CRP POCT devices for LRTI in primary care demonstrated a reduction in immediate antibiotic prescribing of 20% (without affecting symptom resolution or hospital admissions), however, this reduction was not maintained at 28 day follow-up, and there was a significant increase in re-attendance.^28^ In this study, re-attendance rates were similar to that seen in previous studies of LRTI,^29^ and it was encouraging to see that re-attendance was actually lower among patients who were not prescribed immediate antibiotics following FebriDx^®^ testing. Only one small retrospective study at a single GP practice has previously studied the use of FebriDx^®^ in UK primary care, involving 21 patients (mean age 46 years).^12^ Of the 12 patients presenting with suspected bacterial aetiology, clinical management was reportedly altered in 67% (8/12) who were not subsequently prescribed antibiotics. No data was reported on re-attendance rates, test failure rate, diagnostic accuracy or ease-of-use.^12^

The low rate of MxA detection in our study was similar to that seen in recent studies of FebriDx^®^ for LRTI in secondary care (14%–16%). A lower rate of CRP detection meant that the rate of negative results was higher in our study compared with these studies (20%–49%),^13,16,18–20^ which may be due to differences in our primary care patient cohort (including lower disease severity). It is also worth noting that nearly half of our participants presented with over a week of symptoms, and given that MxA is known to rise very early in viral infection (with a half-life of 2 days), this may have also contributed to the low MxA detection rate.^11^ In our study, 46% (24/52) of those with positive test results had detectable MxA, either alone or combined with CRP. When considering the beneficial effect of MxA testing, if only CRP testing were available, we can estimate that all 19 (an additional 13) participants with a combined MxA/CRP result would have been prescribed antibiotics and 32% of those with a MxA-only positive result (an additional two participants). Therefore, MxA testing is likely to have led to an extra 10% (15/155) reduction in antibiotic prescribing over CRP testing alone. A joint MxA-CRP result may indicate a viral infection with an associated inflammatory response, or a ‘dual’ infection/bacterial superinfection. Thorough clinical assessment and safety-netting is therefore key, but unless pneumonia is suspected, such patients can usually be managed safely without antibiotics.^23^

This is the first study to evaluate FebriDx^®^ ease-of-use. As a single-use, hand-held test, FebriDx^®^ offers advantages over many current POCT devices that require an additional desktop analyser, especially in the primary care setting where clinicians usually work in single rooms.^3,5,6,12,30^ There are likely to be technical challenges initially, and users need experience before they can use test reliably. Understanding usability issues is important as they will impact on adoption into routine care,^3,5,6,31^ and we have conducted a qualitative process analysis alongside this study, which we report separately. The test failure rate was higher than the 0%–5% rate reported in recent UK studies of FebriDx^®^ as an emergency department triage tool,^15,16,18^ possibly due to a higher degree of operator error (at least initially) compared with users in emergency departments who perform a higher number of tests. Longer run-in periods in those studies may have allowed users to gain confidence before data collection.^15,16,18^

These results support a funding application for a fully powered trial to assess the impact of using FebriDx^®^ to guide antibiotic prescribing for LRTI in primary care. A future trial should also assess impact on symptoms and safety (including re-attendance) and cost-effectiveness, particularly as costs of implementation are a key barrier to routine adoption of POCT.^3,5,26^ At approximately £12.75 per FebriDx^®^ test (shelf life of 18 months), the overall cost is similar to CRP POCT cartridges, but without any additional up-front or maintenance costs.^30,32^ It is also important to assess clinician and patient views on FebriDx^®^ to explore feasibility and usability in more depth. This includes experience of reading/interpreting results and communicating these to patients, as well as overall patient satisfaction and the feasibility of integrating FebriDx^®^ into real-life practice. We will explore these in our qualitative interview sub-study (reported separately).

Future studies should also assess the role of FebriDx^®^ for upper respiratory tract infections for which antibiotics are commonly prescribed (such as sinusitis), as well as the impact on antiviral prescriptions. It is also important to consider the implementation of FebriDx^®^ and other POCT devices within the wider primary care system. Delivery of primary care in the UK is evolving, and involves a diverse range of allied health care professionals, including dedicated LRTI clinics at primary care network level. POCT testing in such clinics may be more effective and sustainable than opportunistic use in a traditional clinic setting. Future research should also consider assessing the use of FebriDx^®^ in other settings, such as nursing homes and out-of-hours urgent care (settings associated with the highest rates of inappropriate antibiotic prescribing^33,34^), as well as community pharmacies, considering the expanding role of POCT and antibiotic prescribing in this setting.^35^

Finally, the ‘real world’ diagnostic accuracy of FebriDx^®^ in the primary care setting should be assessed, as data from secondary care cannot necessarily be extrapolated as the sensitivity of a test may vary by disease severity (spectrum bias). Future analyses should also explore differences in those presenting in the first week of illness (for which FebriDx^®^ is formally marketed) compared with those presenting after 7 days. Assessment of MxA diagnostic accuracy may be confounded by a low viral load (i.e. low level viral RNA can be detected for prolonged periods after the host immunological response has resolved), as well as certain viruses (including Rhinovirus) that are largely confined to the respiratory tract and may not be associated with a detectable MxA response.^36^ Assessing accuracy for bacterial detection is also challenging due to the lack of reference standard and inability to distinguish colonizing organisms from pathogens, and so would likely rely on clinical adjudication alongside laboratory biomarkers and pathogen detection.^20^

### Conclusions

Use of FebriDx^®^ may reduce unnecessary antibiotic use in patients with LRTI. These findings need confirming in an adequately powered RCT, and our study has found good evidence for the feasibility of conducting such a trial.

## Supplementary Material

dkae127_Supplementary_Data

## Transparency declarations

T.W.C. was an invited expert commentator on the FebriDx NICE Medtech briefing, August 2020. T.W.C. has received equipment and consumables at discount or free of charge for the purposes of independent research from BioFire diagnostics, BioMerieux, QIAGEN and Sherlock Biosciences. He has received speaker fees, honoraria and travel re-imbursement from BioFire diagnostics, BioMerieux, QIAGEN and Janssen. He has received consultancy fees from BioMerieux, QIAGEN, Cepheid, Roche, Janssen and Synairgen research. He has been a member of advisory boards for Cepheid, Roche, Janssen, Shiongi, Sanofi and Seqirus and has acted as a member of independent data monitoring committees for trials sponsored by Roche. T.W.C. has acted as an independent scientific adviser to UK DHSC on rapid diagnostics as part of the COVID-19 pandemic response. All other authors declare no conflicts of interest, including no support from any organization for the submitted work, no financial relationships with any organizations that might have an interest in the submitted work in the previous 3 years, and no other relationships or activities that could appear to have influenced the submitted work. FebriDx^®^ devices for the study were purchased from Lumos Diagnostics, who also provided training to the study team on their use, but had no role in the study design, conduct of the study, data interpretation or write-up.

## Supplementary data

Figures S1 and S2 and Tables S1–S7 are available as Supplementary data at *JAC* Online.
