# Supplementary material for: Use of the FebriDx® host-response point-of-care test may reduce antibiotic use for respiratory tract infections in primary care: a mixed-methods feasibility study
Source: J Antimicrob Chemother. 2024 May 6;79(6):1441–9. doi: 10.1093/jac/dkae127 (PMC11144485; doi:10.1093/jac/dkae127)
Supplement: dkae127_Supplementary_Data [file dkae127_supplementary_data.docx]

**Supplementary data**

1. **Tables**

**Supplementary Table 1: Summary of recruiting GP practice sites**

|  | **Practice 1** | **Practice 2** | **Practice 3** | **Practice 4** | **Practice 5** | **Practice 6** | **Practice 7** | **Practice 8** | **Practice 9** |
| --- | --- | --- | --- | --- | --- | --- | --- | --- | --- |
| **Domain** |  |  |  |  |  |  |  |  |  |
| Registered patients (N) * | 16,168 | 21,452 | 10,888 | 18,861 | 34,312 | 58,340 | 14,057 | 37,871 | 9,624 |
| Deprivation decile * † | 9 | 9 | 10 | 9 | 7 | 7 | 10 | 4 | 10 |
| Ethnicity (proportion of white ethnic groups) | 98.4% | 97.2% | 97.3% | 97.2% | 97.1% | 95.8% | 98.3% | 94.5% | 97.7% |
| Number of patients recruited | 61/162 (38%) | 4/162 (2%) | 29/162 (18%) | 38/162 (23%) | 9/162 (6%) | 7/162 (4%) | 7/162 (4%) | 1/162 (1%) | 6/162 (4%) |

* Data obtained from the Public Health England National General Practice Profiles (accessed 15/05/2023)

† Index of Multiple Deprivation (IMD) decile (1 = most deprived, 10 = least deprived)

**Supplementary Table 2: Change to antibiotic prescription decision before and after FebriDx ® testing**

| **Prescribing decision** | **N (%)** | **Change** | **Total N (%)** |
| --- | --- | --- | --- |
| Immediate to none | 29/155 (19%) | REDUCTION | 73 (47%) |
| Delayed to none | 38 (25% |  |  |
| Immediate to delayed | 6 (4%) |  |  |
| No change | 70 (45%) | No change | 70 (45%) |
| Delayed to immediate  None to delayed  None to immediate | 10 (7%)  1 (1%)  1 (1%) | INCREASE | 12 8%) |

**Supplementary Table 3: Re-consultation rates, split by post-test antibiotic prescription plan**

| **Type of healthcare contact** | **All patients** | **No antibiotics** | **Delayed antibiotics** | **Immediate antibiotics** |
| --- | --- | --- | --- | --- |
| Any | 35/155 (23%) | 17/86 (20%) | 1/17 (6%) | 17/52 (33%) |
| In-hours primary care | 30/155 (19%) | 14/86 (16%) | 1/17 (6%) | 15/52 (29%) |
| Out-of-hours primary care | 7/155 (5%) | 4/86 (5%) | 0/17 (0%) | 3/52 (6%) |
| ED admission | 3/155 (2%) | 2/86 (2%) | 0/17 (%) | 1/52 (2%) |
| Hospital admission | 3/155 (2%) | 2/86 (2%) | 0/17 (%) | 1/52 (2%) |

**Supplementary Table 4: Re-consultation rates split by antibiotic prescribing decision before and after FebriDx ® testing**

| **Pre-test Prescribing decision** | **Post-test Prescribing decision** | **Re-consultation rate (%)** |
| --- | --- | --- |
| Immediate antibiotics | Immediate antibiotics | 13/41 (32%) |
| Immediate antibiotics | Delayed antibiotics | 0/6 (0%) |
| Immediate antibiotics | No antibiotics | 8/29 (28%) |
| Delayed antibiotics | Immediate antibiotics | 4/10 (40%) |
| Delayed antibiotics | Delayed antibiotics | 1/10 (10%) |
| Delayed antibiotics | No antibiotics | 3/38 (3%) |

**Supplementary Table 5: Comparison of FebriDx ® MxA positivity compared to reference standard of PCR positivity for identification of viral infection (N=28)**

|  | **MxA positive** | **MxA negative** |
| --- | --- | --- |
| **PCR positive for viral infection** | 7 | 10 |
| **PCR negative for viral infection** | 1 | 10 |

**Supplementary Table 6: Results of PCR testing for participants who provided nasopharyngeal swabs (N=28)**

| **PCR result** | **N (%)** |
| --- | --- |
| Negative | 11/28 (39%) |
| Influenza B | 2/28 (7%) |
| Metapneumovirus | 2/28 (7%) |
| Parainfluenza 3 | 5/28 (18%) |
| Rhinovirus | 5/28 (18%) |
| SARS-CoV-2 | 3/28 (11%) |

**Supplementary Table 7: Open-text comments from the ease-of-use questionnaire**

| Subject 1 | *“The device was easy to use and user friendly. The interpretation/results with whether there was a faint line and how faint was acceptable…was difficult at first…once we had used the system a few times it was easier to determine”* |
| --- | --- |
| Subject 10 | *“Great concept and could be a fantastic tool in everyday practice, but, the device needs to be easier and quicker to use in a 10-minute consultation for it to be practical”* |
| Subject 11 | *“It was a great system apart from it frequently being challenging to get the blood to transfer out of the collection tube”* |
| Subject 12 | *“Difficulty at times with blood flowing from collection tube into test kit itself…if possible for time from test to result to be shorter would make easier to integrate into clinic”* |
| Subject 14 | *“Lines were too faint”* |
| Subject 15 | *“Definitely use of febrix becomes easier the more you do. There is a knack to it but this became apparent after about 5 uses”* |

**2. Figures**

**Supplementary figure 1: FebriDx ® Ease-of-use questionnaire**

Many thanks for completing this short survey.

The questions below are taken from the System Usability Scale (SUS) questionnaire. The ‘system’ in these questions refers to conducting FebriDx testing in a routine general practice setting (i.e. taking the finger-prick blood sample, applying it to the device, and achieving a result).

We are not asking about wider aspects of using the test (discussing use of the test and findings with patients, patient-flow, ordering/storing tests, etc.). These aspects will be covered in the interviews.

**Each criteria is graded on a 5-point scale from strongly agree to strongly disagree**

1.     I think that I would like to use this system frequently.

2.     I found the system unnecessarily complex.

3.     I thought the system was easy to use.

4.     I think that I would need the support of a technical person to be able to use this system.

5.     I found the various functions in this system were well integrated.

6.     I thought there was too much inconsistency in this system.

7.     I would imagine that most people would learn to use this system very quickly.

8.     I found the system very cumbersome to use.

9.     I felt very confident using the system.

10.   I needed to learn a lot of things before I could get going with this system.

**How many FebriDx tests have you performed?**

0-5

6-10

11-15

16-20

21+

**Any further comments?**

**Supplementary figure 2: Effect of testing on clinician confidence in the need for antibiotics**
